# The prevalence of headache disorders in children and adolescents in Benin: a schools-based study

**DOI:** 10.1186/s10194-024-01843-x

**Published:** 2024-08-22

**Authors:** Mendinatou Agbetou Houessou, Thierry Adoukonou, Willy Tchuenga Fokom, Nelly Dovoedo, Tayyar Şaşmaz, Fatma Bozdağ, Derya Uluduz, Timothy J. Steiner

**Affiliations:** 1 Neurology Department, University of Parakou, Parakou, Benin; 2 National Teaching Hospital HKM of Cotonou, University of Abomey-Calavi, Cotonou, Benin; 3 Department of Public Health, Mersin University School of Medicine, Mersin, Turkey; 4 Department of Public Health, Division of Occupational Medicine, Faculty of Medicine, Gazi University, Ankara, Turkey; 5 Neurology Department, Cerrahpaşa School of Medicine, Istanbul University, Istanbul, Turkey; 6 Department of Neuromedicine and Movement Science, Norwegian University of Science and Technology, Edvard Griegs gate, Trondheim, Norway; 7 Department of Neurology, University of Copenhagen, Copenhagen, Denmark; 8 Division of Brain Sciences, Imperial College London, London, UK

**Keywords:** Child and adolescent headache, Migraine, Tension-type headache, Medication-overuse headache, Undifferentiated headache, Epidemiology, Prevalence, Schools-based study, Benin, Sub-Saharan Africa, Global Campaign against Headache

## Abstract

**Background:**

A global schools-based programme within the Global Campaign against Headache is estimating the burden of headache in children (6–11 years) and adolescents (12–17 years), cluster-sampling the world by conducting national studies in all world regions. Its purpose is to complement population-based studies in adults, adding to knowledge of the burden of headache and informing educational and health policies. This study in Benin was the third in the programme from sub-Saharan Africa (SSA).

**Methods:**

We followed the generic protocol for the global study. In a cross-sectional survey, the child and adolescent versions of the Headache-Attributed Restriction, Disability, Social Handicap and Impaired Participation (HARDSHIP) structured questionnaire were administered to pupils within their classes in 16 schools selected from across the country to be representative of its diversities. Headache diagnostic questions were based on ICHD-3 criteria but for the inclusion of undifferentiated headache (UdH).

**Results:**

Very large proportions of pupils were absent on the survey days. The sampled population defined by class registers totalled 11,802 pupils, of whom only 2,488 were present. A further 193 pupils (or their parents) declined the survey. The surveyed sampled (*N* = 2,295; males 1,156 [50.4%], females 1,139 [49.6%]) included 1,081 children (47.1%) and 1,214 adolescents (52.9%), with a non-participating proportion (193/2,488) of 7.8%. Headache ever was reported by 97.3% of the sample. Age- and gender-adjusted 1-year prevalences, according to responses given, were 53.4% for migraine (almost three quarters of this being probable migraine), 21.3% for tension-type headache, 8.2% for UdH, 1.0% for probable medication-overuse headache (pMOH) and 2.6% for other headache on ≥ 15 days/month (H15+). Both pMOH and other H15 + were substantially more prevalent among adolescents.

**Conclusion:**

The finding for migraine is anomalous, but, within this series of studies, the same was found in Zambia and similar in Ethiopia, both in SSA. While many cases identified as probable migraine, especially among children, might better have been diagnosed as UdH, the true prevalence of migraine almost certainly exceeds 21%. Regardless of diagnosis, headache is very common among children and adolescents in Benin. The study sounds an alarm with regard to pMOH as a developing problem pre-adulthood.

## Introduction

In adults, headache disorders are the third-highest cause of disability worldwide [1, 2]. Migraine, tension-type headache (TTH) and the group of headache disorders characterised by headache on ≥ 15 days/month (H15+), including medication-overuse headache (MOH), all contribute substantially to population ill health among adults [3]. All of these are already common among children and adolescents [4–10], while undifferentiated headache (UdH) is also prevalent in these age groups. UdH is believed to be expressions of migraine or TTH by the immature brain [4] while meeting International Classification of Headache Disorders (ICHD) diagnostic criteria [11] for neither.

Headache disorders are of amplified importance in these young age groups because, while portending lifelong health loss, they also interrupt education, potentially jeopardising life’s opportunities with cumulative lifelong disadvantage [12].

This study continued the series of studies [4–10] that constitute the global schools-based programme [13] within the Global Campaign against Headache, directed by *Lifting The Burden* (LTB) [14–16]. The programme’s ultimate purpose is to estimate the burden of headache in children (6–11 years) and adolescents (12–17 years) worldwide, complementing population-based studies in adults [16]. The programme uses standardised methodology and instruments [13], conducting national studies in all world regions to cluster-sample the world [16, 17]. This study, in Benin, a Francophone West African country, was the third within the programme from sub-Saharan Africa (SSA), following those in Ethiopia in the east [8] and Zambia in the south [9].

Of Benin’s 13 million population, 42% are aged under 16 years [18]. Benin was upgraded by the World Bank in 2020 from low-income status to lower-middle income [19], but its literacy rate nevertheless remains low (about 42%; males 54%, females 31% [20]). Urbanisation at the time of the survey was 48% [20]. In countries in such circumstances, schools-based studies are the only viable way, logistically and economically, of reaching these age groups [13, 17]. A key factor, however, is the proportion of those of school age who are enrolled in school. Education is now free in Benin, and primary education is universal, but secondary school enrolment is < 70% among males and < 60% among females [21]. A schools-based study in this country was therefore expected to provide nationally representative data for children, but have potential biases among adolescent samples, particularly females.

The aims of this study were to estimate, separately among children and adolescents, the prevalences (reported here) and the attributed burdens (to be reported later) of headache overall and of each of the various headache types. Its purposes were to inform local health and educational policies, and to add to knowledge of the global headache burden.

## Methods

The study was a cross-sectional survey following the generic protocol [13], conducted by self-completed structured questionnaires administered in schools selected to be representative of Benin’s diversities.

### Ethics and approvals

The study was approved by the Ethics Committee of the University of Parakou (ref: 0265/CLERB-UP/P/SP/R/SA).

The survey was explained to school managers and teachers of selected schools, whose agreement to participate was required. Information forms describing the study’s nature and purposes were given to all intended participants, for themselves and their parents. Pupils’ consents were required for participation. In keeping with the terms of ethics approval, parents were asked to notify the school should they object, and were presumed to be in agreement if they did not.

### Sampling and recruitment

The study was conducted from April to June 2019.

**Fig. 1:**
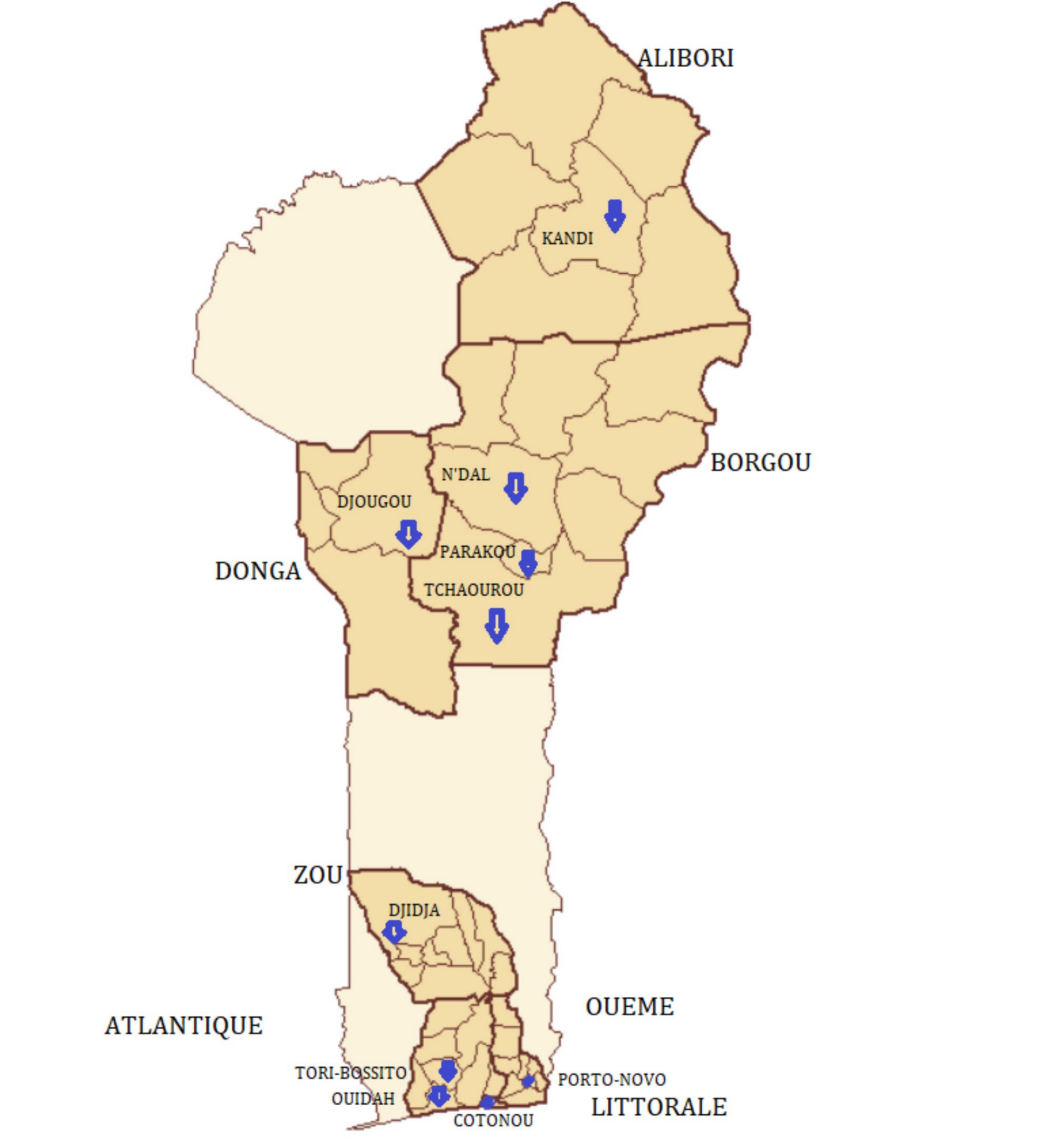
The sampled areas of Benin

We sampled in three stages, aiming to match the country’s regional and socioeconomic diversities within the sample. First, we randomly selected, by drawing lots, three of the country’s four departments in the north (Alibori, Borgou, Donga) and four of the eight in the south of Benin (Atlantique, Littoral, Ouémé, Zou) (Fig. 1). From these we purposively selected 16 schools, eight urban and eight rural, and at least two from each department, in order to achieve both urban-rural and public-private parity with respect to the country as a whole. Finally, we invited all pupils aged 6–17 years, from all classes, who were present on the survey days and for whom no parental objection had been registered, to participate.

Those declining, as well as those whose parents had objected, were counted as non-participants, but those who were absent were not, being unavailable for inclusion.

We aimed for *N* > 2,000 as recommended by guidelines [17].

### Survey instruments

We employed the child and adolescent versions of LTB’s Headache-Attributed Restriction, Disability, Social Handicap and Impaired Participation (HARDSHIP) structured questionnaire [13], translated into West African French according to LTB’s translation protocol for hybrid documents [22]. These modular instruments, designed for self-completion under supervision, incorporated demographic enquiry, headache screening and diagnostic questions based on ICHD-3 criteria [11] and enquiries into components of headache-attributed burden [13]. The timeframes of enquiry were the preceding 4 weeks (28 days) and one week, except for the module asking specifically about headache yesterday (HY).

Pupils completed their questionnaires in class under the supervision of the teacher or an investigator. These questionnaires did not identify participants, and data collection was anonymous except to the extent that younger children, and those unable to read well, were given necessary assistance in understanding and appropriately answering the questions.

Additional questionnaires enquiring into relevant school variables were completed by teachers of the schools [13].

### Diagnoses

Diagnoses were made algorithmically according to HARDSHIP methodology [12, 13]. H15+ (i.e., headache reported on ≥ 14 days in the preceding 28 days) was first identified, and categorised according to reported frequency of acute medication use into probable MOH (pMOH: use on ≥ 14 days/28 days) or “other H15+” (use on 0–13 days/28 days). To all other reported headaches, the algorithm first applied criteria for UdH (mild intensity and usual duration < 1 h [4]) and then the ICHD-3 criteria for definite migraine, definite TTH, probable migraine and probable TTH in this strict order, recognising the hierarchy of ICHD [11]. Remaining cases were recorded as unclassified.

### Data management and entry

All survey forms were stored securely in the Neurology Department of the University of Parakou. All data were entered twice into SPSS, independently by two investigators (MAA and WTF). The two datasets were compared for discrepancies, which were resolved by reference to the source data.

### Analysis

Analyses were performed at University of Mersin. The analytical methods were those adopted by all similar Global Campaign studies [4–10], maintaining ease of comparison.

We categorised schools by location (urban, semi-rural or rural) and, as a socio-economic indicator, by teachers’ estimates of proportions of pupils coming from low-income homes (< 0.25, 0.25–0.49, 0.5–0.75 [for simplicity we refer to these categories as “high-income”, “middle-income” and “low-income”]). We also characterised schools by proportions of pupils living at distances of > 1 h’s travel time.

We used descriptive statistics to present means and standard deviations (SDs) of continuous variables and proportions (%) with 95% confidence intervals (CIs) of categorical data. We used chi-squared or one-sample proportion tests to evaluate differences between groups. We estimated prevalences of each headache type as proportions (%), and adjusted the crude values for gender and age using official population statistics for Benin [18]. In these analyses, in accordance with published guidelines [17], definite and probable migraine were combined, as were definite and probable TTH. To show associations with demographic variables, we first used bivariate analysis with odds ratios (ORs), then multivariate logistic regression analysis with adjusted ORs (AORs), entering gender, age group, school location and income category into the multivariate model. Mean headache frequencies (days/month) were calculated to support estimates of predicted headache yesterday.

We considered *p* < 0.05 to be significant.

## Results

Once sampling commenced, it became clear that very large proportions of pupils were absent on the survey days, and that this was not unusual in Benin. The sampled population was defined by the class registers for all classes included in the survey, totalling 11,802 pupils. Of these, only 2,488 were present for the survey, with 9,314 unavailable for sampling. A further 193 pupils (or their parents) declined; therefore, *N* totalled 2,295 (children 1,081 [47.1%], adolescents 1,214 [52.9%]; males 1,156 [50.4%], females 1,139 [49.6%]). The non-participating proportion was 7.8% (193/2,488). Despite the large proportion absent, we achieved similar numbers of each gender and reasonably similar numbers of children and adolescents. Mean age of the sample was 12.1 ± 3.1 years (median 12).

In Table 1 we categorise participants according to school variables, using data provided by the teachers. Almost half (47.1%) attended urban schools, and 52.9% attended semi-rural or rural schools, well reflecting Benin’s 48% urbanisation at the time [20]. These proportions were matched by those travelling for > 1 h to reach school. One third of participants (34.1%) attended schools where > 50% of pupils were from low-income homes.

**Table 1:** Participating pupils categorised according to school variables (from teachers’ questionnaires) (*N* = 2,295)

Variable	Pupils	
	*n*	%
School location		
urban semi-rural rural	1,081 365 849	47.1 15.9 37.0
Categorisation according to estimated proportion of pupils travelling for > 1 h		
< 0.25 0.25–0.5 0.5–0.75	1,081 433 781	47.1 18.9 34.0
Income category according to estimated proportion of pupils from low-income homes		
< 0.25 (“high-income” school) 0.25–0.5 (“middle-income” school) 0.5–0.75 (“low-income” school)	515 999 781	22.4 43.5 34.1

### Headache

Headache ever was reported by 2,234 pupils (crude lifetime prevalence: 97.3%), and headache in the preceding year by 2,032 (crude 1-year prevalence: 88.5%; gender- and age-adjusted: 88.6%). Observed and adjusted prevalences of each headache type are shown in Table 2.

**Table 2:** Crude (observed) 1-year prevalences of all headache and each headache type, overall and according to demographic variables, and gender- and age-adjusted prevalences (*N* = 2,295)

	All headache (*n* = 2,032)	Migraine (*n* = 1,196)	Tension-type headache (*n* = 500)	pMOH (*n* = 27)	Other headache on ≥ 15 d/m (*n* = 64)	UdH (*n* = 194)
**Crude prevalences** (% [95% CI])						
Overall	88.5 [87.2–89.8]	52.1 [50.1–54.1]	21.8 [20.1–23.5]	1.2 [0.8–1.7]	2.8 [2.1–3.5]	8.4 [7.3–9.5]
Gender						
male (*n* = 1,156) female (*n* = 1,139)	86.7 [84.7–88.7] 90.4 [88.7–92.1]	51.5 [48.6–54.4] 52.8 [49.9–55.7]	21.1 [18.8–23.5] 22.5 [20.1–24.9]	0.8 [0.3–1.3] 1.6 [0.9–2.3]	1.7 [1.0-2.5] 3.9 [2.8-5.0]	9.5 [7.8–11.2] 7.4 [5.9–8.9]
Age group (years)						
6–11 (*n* = 1,081) 12–17 (*n* = 1,214)	89.1 [87.2–91.0] 88.1 [86.3–89.9]	57.8 [54.9–60.7] 47.0 [44.2–49.8]	19.7 [17.3–22.1] 23.6 [21.2–26.0]	0.5 [0.1–0.9] 1.8 [1.1–2.6]	2.0 [1.2–2.8] 3.5 [2.5–4.5]	7.3 [5.8–8.9] 9.5 [7.9–11.2]
School income category*						
high (*n* = 515) middle (*n* = 999) low (*n* = 781)	95.0 [93.1–96.9] 86.9 [84.8–89.0] 86.4 [84.0-88.8]	60.6 [56.4–64.8] 54.1 [51.0-57.2] 44.0 [40.5–47.5]	16.3 [13.1–19.5] 20.4 [17.9–22.9] 27.1 [24.0-30.2]	2.5 [1.2–3.9] 0.3 [0.0-0.6] 1.4 [0.6–2.2]	4.3 [2.6–6.1] 1.6 [0.8–2.4] 3.3 [2.1–4.6]	7.4 [5.1–9.7] 9.0 [7.2–10.8] 8.5 [6.5–10.5]
School locality						
urban (*n* = 1,081) semi-rural (*n* = 365) rural (*n* = 849)	87.1 [85.1–89.1] 94.0 [91.6–96.4] 88.0 [85.8–90.2]	53.3 [50.3–56.3] 66.3 [61.5–71.2] 44.5 [41.2–47.8]	17.7 [15.4–20.0] 11.5 [8.2–14.8] 31.4 [28.3–34.5]	1.5 [0.8–2.2] 3.0 [1.3–4.8] 0.0 [0.0–0.0]	3.1 [2.1–4.1] 5.8 [3.4–8.2] 1.2 [0.5–1.9]	8.8 [7.1–10.5] 7.1 [4.5–9.7] 8.6 [6.7–10.5]
**Gender and age-adjusted prevalences** (% [95% CI])						
Overall	88.6 [88.2–89.0]	53.4 [52.8–54.1]	21.3 [20.8–21.8]	1.0 [0.9–1.1]	2.6 [2.4–2.8]	8.2 [7.8–8.6]

pMOH: probable medication-overuse headache; d/m: days/month; UdH: undifferentiated headache; CI: confidence interval; * see text or Table 1 for explanation. Tables 4 and 5 provide odds ratios and adjusted odds ratios for these comparisons

According to responses given, migraine, with an adjusted 1-year prevalence of 53.4%, accounted for 60.3% of all reported headache. Almost three quarters (72.3%) of cases labelled as migraine were of probable migraine. TTH (21.3%) and UdH (8.2%) were less common (Table 2). H15 + affected 3.6% of participants, including 1.0% with pMOH. There were 51 headache cases (2.2%) remaining unclassified.

We looked at the responses among those with headache driving the diagnosis of migraine: headache characteristics, and symptoms specific to migraine reported as usually accompanying headache (nausea, vomiting, photophobia, phonophobia) (Table 3). We also considered headache duration (more or less than 2 h). Only 36.5% of participants with headache of any type reported usual durations of > 2 h, the ICHD threshold for migraine in children [11]. Of those given the diagnosis of probable migraine (*n* = 865), only 159 (18.4%) reported usual durations of > 2 h, while 357 (41.3%) reported < 1 h. With the exception of unilaterality, all migraine-like characteristics were reported by well over half of participants with headache of any type. As for accompanying symptoms, 420 (48.6%) of those diagnosed with probable migraine reported nausea (*versus* 35.9% among all with headache), 330 (38.2%) reported vomiting (*versus* 29.1%), 612 (70.8%) reported photophobia (preference for dark) (*versus* 51.1%) and 820 (94.8%) reported phonophobia (preference for quiet) (*versus* 87.8%). None of these stood out as a principal driver.

**Table 3:** Responses among those with headache likely to drive the diagnosis of migraine [11] (*N* = 2,032)

Headache characteristics	Response *n* (%)	
	Yes	No
Duration > 2 h	742 (36.5)	1,290 (63.5)
Moderate or severe intensity	1,620 (79.7)	412 (20.3)
Unilateral	398 (19.6)	1634 (80.4)
Throbbing	1,386 (68.2)	646 (31.8)
Aggravated by routine activity	1,475 (72.6)	557 (27.4)
Causing avoidance of routine activity	1,259 (62.0)	773 (38.0)
**Accompanying symptoms**		
Nausea	730 (35.9)	1,302 (64.1)
Vomiting	591 (29.1)	1,441 (70.9)
Photophobia	1,039 (51.1)	993 (48.9)
Phonophobia	1,785 (87.8)	247 (12.2)

### Demographic associations

These are illustrated in Tables 2, 4 and 5. Gender-related differences were small, and in multivariate analysis significant only for other H15+ (more common among females: AOR = 2.2). Migraine was, apparently, less prevalent among adolescents (AOR = 0.7), but TTH (AOR = 1.5), pMOH (AOR = 4.2) and UdH (AOR = 1.5) were all more common, with no difference in headache overall. As a proportion of all headache, UdH increased from 8.2% among children to 10.8% among adolescents. The most notable association was of pMOH with adolescence (AOR = 4.2). Other H15 + was also reportedly more prevalent among adolescents (3.5% versus 2.0% [Table 2]), but this was not significant in multivariate analysis (Table 5).

There was a positive association between school-income category and migraine, evident in bivariate analysis (Table 4) and emphasised in multivariate analysis (Table 5). A negative association with TTH disappeared in the latter (Table 5). Associations with school locality were also dissimilar between migraine and TTH: semi-rural school location (but not rural) substantially increased the odds of migraine (aOR = 2.65 in comparison with urban); rural location (but not semi-rural) substantially increased the odds of TTH (aOR = 2.5) (Table 5).

**Table 4:** Bivariate analyses of headache type *versus* demographic variables (*N* = 2,295)

Variable	Migraine (*n* = 1,196)	Tension-type headache (*n* = 500)	pMOH (*n* = 27)	Other headache on ≥ 15 d/m (*n* = 64)	UdH (*n* = 194)
	Odds ratio [95% CI]				
Gender					
male (*n* = 1,156) female (*n* = 1,139)	reference 1.05 [0.9–1.2]	reference 1.1 [0.9–1.3]	reference 2.05 [0.9–4.6]	reference **2.3** [1.3–3.9]^2^	reference 0.8 [0.6–1.02]
Age group (years)					
6–11 (*n* = 1,081) 12–17 (*n* = 1,214)	reference **0.65** [0.55–0.8]^3^	reference **1.3** [1.03–1.5]^1^	reference **4.0** [1.5–10.5]^2^	reference **1.7** [1.02–2.9]^1^	reference 1.3 [0.98–1.8]
School income category*					
high (*n* = 515) middle (*n* = 999) low (*n* = 781)	reference **0.8** [0.6–0.95]^2^ **0.5** [0.4–0.6]^3^	reference 1.3 [0.99–1.7] **1.9** [1.4–2.5]^3^	reference **0.1** [0.03–0.4]^3^ 0.55 [0.25–1.2]	reference **0.4** [0.2–0.7]^2^ 0.8 [0.4–1.4]	reference 1.2 [0.8–1.9] 1.2 [0.8–1.8]
School locality					
urban (*n* = 1,081) semi-rural (*n* = 365) rural (*n* = 849)	reference **1.8** [1.35–2.2]^3^ **0.7** [0.6–0.8]^3^	reference **0.6** [0.4–0.9]^2^ **2.1** [1.7–2.65]^3^	reference 2.1 [0.95–4.5] --	reference **1.9** [1.1–3.4]^2^ **0.4** [0.2–0.8]^2^	reference 0.8 [0.5–1.25] 0.98 [0.7–1.3]

pMOH: probable medication-overuse headache; d/m: days/month; UdH: undifferentiated headache; CI: confidence interval; * see text or Table 1 for explanation; significant values are emboldened: ^1^*p* < 0.05; ^2^*p* < 0.01; ^3^*p* < 0.001

**Table 5:** Multivariate logistic regression analyses of headache type *versus* demographic variables (*N* = 2,295)

Variable	Migraine (*n* = 1,196)	Tension-type headache (*n* = 500)	pMOH (*n* = 27)	Other headache on ≥ 15 d/m (*n* = 64)	UdH (*n* = 194)
	Adjusted odds ratio [95% CI]				
Gender					
male (*n* = 1,156) female (*n* = 1,139)	reference 0.97 [0.8–1.15]	reference 1.2 [0.98–1.5]	reference 1.8 [0.8–4.1]	reference **2.2** [1.3–3.8]^2^	reference 0.8 [0.6–1.03]
Age group (years)					
6–11 (*n* = 1,081) 12–17 (*n* = 1,214)	reference **0.7** [0.6–0.9]^2^	reference **1.5** [1.2–1.9]^2^	reference **4.2** [1.6–11.2]^2^	reference 0.9 [0.5–1.7]	reference **1.5** [1.02–2.1]^1^
School income category*					
high (*n* = 515) middle (*n* = 999) low (*n* = 781)	reference **0.5** [0.4–0.7]^3^ **0.4** [0.3–0.5]^3^	reference 1.3 [0.9–1.75] 0.9 [0.6–1.5]	† † †	reference **0.45** [0.2–0.9]^1^ 2.6 [0.7–9.8]	reference 1.5 [0.95–2.3] 1.1 [0.6–2.2]
School locality					
urban (*n* = 1,081) semi-rural (*n* = 365) rural (*n* = 849)	reference **2.65** [1.9–3.6]^3^ 1.2 [0.9–1.6]	reference 0.7 [0.4–1.02] **2.5** [1.8–3.6]^3^	reference 2.5 [1.1–5.5] --	reference 1.06 [0.35–3.2] **0.15** [0.04–0.6]^2^	reference 0.8 [0.5–1.4] 1.01 [0.6–1.7]

pMOH: probable medication-overuse headache; d/m: days/month; UdH: undifferentiated headache; CI: confidence interval; * see text or Table 1 for explanation; † variable excluded in the logistic regression model; significant values are emboldened: ^1^*p* < 0.05; ^2^*p* < 0.01; ^3^*p* < 0.001

### Headache yesterday (HY)

Among those with any headache in the last year, HY was reported by 430 pupils (21.2%; 18.7% of the total sample) (Table 6). Females (25.3%) reported HY more than males (16.9%; *p* < 0.001), and adolescents (26.1%) more than children (15.7%; *p* < 0.001).

**Table 6:** Proportions reporting headache yesterday, and predicted proportions*, overall and by headache type

Headache type	Headache yesterday		
	Reported proportion *n* (%)	Predicted proportion	
		Mean reported headache frequency (F) (days/4 weeks)	Predicted headache yesterday* (%)
**Any headache** (*n* = 2,032)	430 (21.2)	4.2 ± 4.4	15.0
**Migraine** (*n* = 1,196)	237 (19.8)	3.8 ± 2.8	13.6
**TTH** (*n* = 500)	102 (20.4)	3.2 ± 2.6	11.4
**pMOH** (*n* = 27)	18 (66.7)	21.4 ± 4.7	76.4
**Other headache on ≥ 15 d/m** (*n* = 64)	30 (46.9)	18.3 ± 3.6	65.4
**UdH** (*n* = 194)	33 (17.0)	3.0 ± 3.1	10.7

*calculated as F/28; TTH: tension-type headache; pMOH: probable medication-overuse headache; d/m: days/month; UdH: undifferentiated headache

Mean headache frequency in the 2,032 reporting any headache was 4.2 days/28 days. Since the observed prevalence of any headache was 88.5% (Table 2), the proportion of all participants expected to have headache on any day was 13.3% (88.5*4.2/28%), rather fewer than the 18.7% actually reporting HY. Table 6 shows that HY was reported more often than predicted by those with episodic headache (migraine: 19.8% *versus* 13.6%; TTH: 20.4% *versus* 11.4%; UdH: 17.0% *versus* 10.7%; factors of 1.4, 1.8 and 1.6 respectively); those with H15+, including pMOH, the greatest contributor proportionately to HY, reported HY rather less than predicted.

## Discussion

This study, in Francophone West Africa, was the third in SSA of the Global Campaign series of schools-based studies, after those in Ethiopia in the east [8] and Zambia in the south [9]. Despite the fact that only one quarter (27%) of registered pupils were present on the survey days – not unusual according to the teachers, but greatly jeopardising representativeness – the sample was reasonably balanced for gender and age distribution and matched Benin’s urban/rural divide. To summarise the findings, headache ever was reported by almost all (97.3%) of the sample, while age- and gender-adjusted 1-year prevalences were 88.6% for all headache and, according to responses given, 53.4% for migraine (probable migraine accounting for almost three quarters of this), 21.3% for TTH, 8.2% for UdH, 1.0% for pMOH and 2.6% for other H15+.

The finding for migraine is obviously anomalous. We analysed the responses that might drive a diagnosis of probable migraine, rather than probable TTH or UdH (Table 3). None stood out in particular, although moderate or severe intensity, throbbing headache, aggravation by routine activity, photophobia and phonophobia were all reported by > 50% of those with any headache.

The diagnostic question set developed for these studies, based on ICHD [11], appear to have worked well in Turkey [4], Austria [5], Lithuania [6], Mongolia [7] and Iran [10]. Intriguingly, however, very high prevalences of migraine were found in both other countries in SSA that have used this same question set: Ethiopia 38.6%, with 22.5% probable [8], and Zambia 53.2%, with 35.7% probable [9] – very close to the findings here.

In other words, this diagnostic vagary is unique (so far) to SSA, with no obvious explanation. It does not appear to lie in translation of questions, or of response options, since these countries have between them used HARDSHIP in seven languages [8, 9]. Structured questionnaires tend to use leading questions, and the last six of those in Table 3 are framed with response options of “no” or “yes” [11]. Pupils in class, asked to choose between these, might feel the latter to be more obliging. The responses with regard to nausea (35.9% “yes”) and vomiting (29.1%), however, give little support to this as a possible explanation. Young people might also be expected to embellish, but, if this influenced their reporting of intensity, it was not evident in duration, which, reportedly, was generally short.

In Zambia, in fact, we noted that duration was reported as ≤ 2 h by nearly three quarters of pupils with headache of any type [9], and the proportion here was also high (63.5%). ICHD puts a lower limit of 2 h to migraine in children [11]. Although this is opinion-based rather than strongly informed by evidence, applying it to probable migraine in this study would push out 706 of the 865 so diagnosed – mostly towards UdH – reducing the estimated prevalence of all migraine to about 21%. This is at the bottom of the range of estimates of migraine prevalence in the series (21.4–27.3% in non-SSA countries [4–7, 10]). It is probably lower than the true prevalence, but reasonable confidence can be attached to it as a minimum estimate.

This uncertainty surrounding diagnosis extended to the association analysis, at least for the episodic headaches. It is unlikely, for example, that migraine was less prevalent among adolescents (AOR 0.7) than children. Since UdH is understood to represent expressions of migraine or TTH by the immature brain [4], an inverse relationship is expected between UdH prevalence and increasing age. This was seen in Turkey [4], Austria [5], Lithuania [6], Mongolia [7] and Iran [10], but not here (or, notably, in Ethiopia [8] or Zambia [9], also SSA countries, and with similar diagnostic uncertainty occurring in both). However, the associations with school-income category and school locality were of interest because they differentiated migraine from TTH, being markedly dissimilar between these. This suggests diagnostic indistinction between migraine and TTH, which would have masked such differences, was not serious. The findings themselves have no obvious explanation, and we doubt the value of speculation.

Unaffected by this issue, and therefore much more reliable, were the estimates of pMOH prevalence, increasing from 0.5% in children to 1.8% (AOR 4.2) among adolescents. This is of concern. In itself it is worrying as an indicator of mismanagement of child and adolescent headache, but, additionally, this trajectory predicts a high adult prevalence of pMOH in Benin – which has, indeed, been found (4.5% [23]). Notably, other H15 + reportedly increased in prevalence from 2.0% in children to 3.5% among adolescents, carrying with it a commensurately rising risk of pMOH.

The study had several strengths: the tested and validated methodology [4], adequate sample size and participating proportion of > 90% [17]. There were limitations also, particularly those relating to the diagnosis of migraine, discussed above. There are general limitations to surveys among young people, being dependent on their understanding and recall, neither wholly reliable among children in particular. These limitations are to a large extent unavoidable. Direct validation of the diagnostic question set, requiring re-interview of a subsample of participants, is not only logistically challenging but also ethically problematic in this age group in a study offering no individual benefit. An additional problem here was the very high level of absenteeism (73%), despite that education is free in Benin [21]. This undermined the careful sampling procedure intended to ensure representativeness, while overriding the initial concern over low secondary school enrolment (< 70% among males and < 60% among females [21]). Nevertheless, the sample was reasonably well balanced for gender, and precisely balanced for urbanisation, and it is most unlikely that the factors contributing to absenteeism were relevant to propensity for headache. The study was not capable of recognising secondary headaches (other than pMOH). Malaria and various infections likely to cause headache are common in SSA, although data collection was completed before the arrival of SARS-CoV-2 (covid). Secondary headaches were unlikely to be reported as short-duration episodes (almost two thirds [63.5%] were described as ≤ 2 h). Secondary headaches might have been among the 2.6% other H15+, but all participating pupils were well enough to be in class.

## Conclusions

Headache is very common in children and adolescents in Benin. The prevalence of migraine almost certainly exceeds 21%. Even with the diagnostic uncertainties, this finding informs educational and health policies in Benin. The study sounds an alarm with regard to pMOH as a developing problem pre-adulthood.

Estimates of attributed burden are more important than prevalence from public-health and educational perspectives. These will follow in a future manuscript.

## Data Availability

The data are held on file at University of Mersin. Once analysis and publications are completed, they will be freely available for non-commercial purposes to any person requesting access in accordance with the general policy of the Global Campaign against Headache.

## Abbreviations

AOR: Adjusted odds ratio
CI: Confidence interval
H15+: Headache on ≥ 15 days/month
HARDSHIP: Headache-Attributed Restriction, Disability, Social Handicap and Impaired Participation (questionnaire)
HY: Headache yesterday
ICHD: International Classification of Headache Disorders
LTB: Lifting The Burden
MOH: Medication-overuse headache
OR: Odds ratio
pMOH: Probable MOH
SD: Standard deviation
SSA: Sub-Saharan Africa
TTH: Tension-type headache
UdH: Undifferentiated headache
